# The need for trust and safety inducing encounters: a qualitative exploration of women’s experiences of seeking perinatal care when living as undocumented migrants in Sweden

**DOI:** 10.1186/s12884-018-1851-9

**Published:** 2018-06-07

**Authors:** My Barkensjö, Josephine T. V. Greenbrook, Josefine Rosenlundh, Henry Ascher, Helen Elden

**Affiliations:** 1 Institute of Health and Care Sciences, Sahlgrenska Academy, University of Gothenburg, Arvid Wallgrens Backe, Box 457, 405 30 Gothenburg, Sweden; 2 School of Psychology, Institute of Psychology, Health, and Society, University of Liverpool, Eleanor Rathbone Building, Bedford Street South, Liverpool, L69 7ZA UK; 3 School of Law, University of Edinburgh, Old College, South Bridge, Edinburgh, Scotland EH8 9YL UK; 4 Department of Public Health and Community Medicine, Sahlgrenska Academy, University of Gothenburg, Medicinaregatan 16A, Box 414, 405 30 Gothenburg, Sweden; 5 Department of Research and Development, Angered Hospital, Gothenburg, Sweden

**Keywords:** Undocumented migrant women, Clinical encounters, Perinatal care, Pregnancy and childbirth, Human rights, Psychological trauma, Vulnerable patients, Migrant health, Social vulnerability, Cultural competence

## Abstract

**Background:**

Studies from around the world have shown that women living as undocumented migrants have limited and deficient access to perinatal care, increasing their risks of both physical and psychological complications during pregnancy and childbirth. Failures to provide equal access to healthcare have been criticized extensively by the United Nations. In 2013, undocumented migrants’ rights to healthcare in Sweden were expanded to include full access to perinatal care. Research surrounding clinical encounters involving women living as undocumented migrants remains largely lacking. The present study aimed to provide a composite description of women’s experiences of clinical encounters throughout pregnancy and childbirth, when living as undocumented migrants in Sweden.

**Methods:**

Taking an inductive approach, qualitative content analysis was implemented. Thirteen women from ten different countries were interviewed. Meaning-units were extracted from the data collected in order to identify emergent overarching themes.

**Results:**

In clinical encounters where healthcare professionals displayed empathic concern and listening behaviours, women felt empowered, acknowledged, and encouraged, leading them to trust clinicians, diminishing fears relating to seeking healthcare services. Conversely, when neglectful behaviour on part of healthcare professionals was perceived in encounters, anxiousness and fear intensified. Vulnerability and distress induced by the women’s uncertain living circumstances were apparent across themes, and appeared exacerbated by traumatic memories, difficulties in coping with motherhood, and fears of deportation.

**Conclusion:**

The present study contributes unique and important knowledge surrounding women’s experience of being pregnant and giving birth when living as undocumented migrants. The overarching findings indicated that the needs of undocumented migrant women were largely similar to those of all expectant mothers, but that due to vulnerabilities relating to their circumstances, flexible and informed care provision is essential. Being knowledgeable on undocumented migrants’ rights to healthcare is vital, as clinical encounters appeared highly consequential to the women’s well-being and help-seeking behaviours. Negative encounters inflicted emotional distress and fear. Contrastingly, positive encounters promoted trust in clinicians, personal empowerment, and relief. Positive clinical encounters could provide rare opportunities to assist an otherwise elusive population at increased risk for both physical and psychological complications, highlighting the crucial need for adherence to ethical principles in clinical practice.

## Background

Undocumented migrants consist of individuals with no legal residency documentation, due to having entered a country illegally, or having remained in violation of the terms of their visa or rejected asylum claims [1]. As with documented migration, this population is motivated by multiple factors, such as asylum-seeking, family reunification, and economic improvement [2]. Whilst predicted numbers are likely to be flawed, due to the elusive nature of this population, in 2011 the number of undocumented migrants residing in Sweden was estimated to be 10.000 to 35.000 [3, 4]. Following the substantial influx in migration in recent years [5], and combined with new, more restrictive asylum legislation, it is likely that these numbers are swiftly increasing. The Swedish Migration Board, has stipulated that an increase of 46.000 to 60.000 new undocumented migrants is to be expected by 2019 [5]. European estimates fluctuate between 1.8 and 3.9 million [6], whereas globally, 50 million migrants are assumed to remain undocumented [7].

A primary goal of the World Health Organizations (WHO) is to realize the enjoyment of the highest attainable standard of health as a fundamental right to every human being, without distinction of race, religion, political belief, and economic or social condition [8]. This is further highlighted in the United Nation’s (UN) *International Covenant on Economic, Social and Cultural Rights* [9], and backed by the World Medical Association (WMA), which explicitly works toward the highest possible standards of ethical behaviour and care by physicians [10]. The International Confederation of Midwives (ICM), which serves as the beacon of midwifery, champions access to perinatal care for all, and holds as its guiding purpose to improve the quality of care for all women, babies, and families worldwide [11].

Within the European Union, rights relating to healthcare access differ considerably between member states, and until recently, undocumented migrants in Sweden have had limited access to healthcare, and thus were charged in full for services needed [1]. This has been criticized by the United Nations, who stated that undocumented migrants constitute one of the most vulnerable groups in society, which human right laws are intended to protect, and that without equal rights to healthcare, they are at increased risk of becoming subject to subpar treatment and discrimination [12]. Whilst free healthcare for undocumented migrants in Sweden used to be provided primarily by non-governmental organizations (NGOs) [13], as of 2013 undocumented migrants have been legally entitled to healthcare and dental care “*that cannot be deferred*”, pregnancy termination, contraceptive counselling, sexual and reproductive care, and perinatal care, as well as access to medical examinations and medicine covered by universal healthcare [14]. The National Board of Health and Welfare [15] has stated that EU citizens residing in Sweden in excess of three months without residency permits should also be considered undocumented migrants, and thus equally entitled to the aforementioned healthcare services. However, the rights of vulnerable EU citizens without an EU health card remains disputed [16].

Both the health professions and the National Board of Health and Welfare have raised concern surrounding the current legislation’s imprecision, as the term ‘*care that cannot be deferred’* is not compatible with medical science, professional medical ethics, or international human rights [17]. The lacking clarity has induced uncertainty, and deficient knowledge amongst healthcare professionals has resulted in failures to deliver services to undocumented migrants [18]. Médecins du Monde [19] found that, amongst undocumented migrants, one of the primary barriers to receiving healthcare services remains the absence of social security numbers.

In Sweden, the majority of women living as undocumented migrants belong to one of two particularly vulnerable groups; rejected asylum-seekers, and overstaying vulnerable EU citizens. Women in the first group have to a high degree escaped war, persecution, sexual and gender based abuse, and/or political oppression. A high rate of psychological traumatization has been found to be present amongst these women [20], and their conditions are often aggravated by fears of being discovered and deported back to the country of persecution. Women in the second group commonly belong to the Roma minority from Eastern Europe and have a long history of discrimination, oppression, and poverty in their countries of origin [21, 22].

Previous research has shown that women living as undocumented migrants use fewer contraceptive methods, and thus, have more unintended pregnancies, at a younger age, are more often non-cohabiting, and have lower incomes than the average pregnant population [23–26]. Furthermore, they have been found to seek perinatal care later, have fewer antenatal visits, and spend fewer days at the hospital postpartum when compared to the general pregnant population [23, 25, 27]. Multiple studies including migrant women (primarily including asylum-seekers and refugees) have shown this cohort to be at increased risk for both pregnancy and delivery related complications, such as anemia, excessive bleeding, fetal distress, stillbirth, pre-term births, and birthing babies small for gestational age (SGA) [28–31]. Moreover, these risks appear aggravated amongst migrant women who do not receive antenatal care during pregnancy [32]. Increased presence of hepatitis B [33], human immunodeficiency virus (HIV), diabetes, sexually transmitted infections, hypertonia, headache, and acute stress have also been observed amongst this cohort, when compared to the general population of pregnant women [34]. An elevated presence of psychopathology has also been observed amongst this group, with post-traumatic stress disorder (PTSD), depression, and anxiety disorders, as well as suicidal ideation and attempts being prevalent [34]. Postpartum depression (PPD) is also more common among migrant women than in native born women, plausibly due to the preceding presence of depression and PTSD substantially increasing the risks for the condition’s development [20].

The rationale guiding the present study was constructed on the empirical indication that undocumented migrant women are in need of qualified and informed care [23, 34], accenting the importance of promoting supporting and trusting clinical relationships at antenatal healthcare centers (ANHC). Nevertheless, studies involving migrants have shown that language barriers resulted in difficulties with booking appointments for healthcare services, and in cases where services were provided, feelings of being ignored, discriminated against, and/or being sent home without receiving the sought-out care have been reported [25, 35–37]. Furthermore, a recent study found that receptionists at healthcare facilities have refused access to women who presented without residency status, and women have also been required to pay in advance when seeking abortions [25]. Midwives have reported experiencing ethical dilemmas due to their limited ability to provide continuous care to women living as asylum-seekers, refugees, and undocumented migrants [34, 38, 39]. Volunteer healthcare staff have reported being concerned about healthcare professionals’ prejudicial attitudes toward migrant women, and a need for NGO assistance in bridging the gap between the inherent marginalized position these women hold and the need for adequately supporting them in perinatal care has been raised in previous findings [38]. These issues are further compounded by the inherent barriers to seeking healthcare services that exist for undocumented migrants, such as fears of being reported to the police, financial restrictions, and lacking knowledge surrounding healthcare systems in host countries [25].

An extremely limited number of empirical explorations have focused on reporting primary experiences of this specific vulnerable population, and observations in the context of perinatal care remain largely lacking. Thus, the present study aimed to provide a composite description of women’s experiences of clinical encounters during pregnancy, childbirth, and the post-partum phase. When living as undocumented migrants in Sweden. The study’s primary objective was to synthesize accounts of both positive and negative engagement on behalf of healthcare professionals in perinatal care, in order to explore how these might impact women known to already be living in an acutely vulnerable situation. The study’s aim and objective were further motivated by the apparent need for identifying crucial stepping-stones, to improve both quality of care and patient safety through the accumulation of new knowledge and awareness on this largely underexplored phenomenon.

## Methods

Qualitative methods are favourable when exploring a largely unknown research field, in the attempt to understand a specific context, and to reach nuanced descriptions surrounding different qualitative aspects of interviewees’ experiences of an observed phenomenon [40–42]. Qualitative approaches also facilitate the surfacing of voices from hidden, vulnerable, and excluded groups in society [42, 43]. Due to the lack of pre-existing knowledge surrounding the phenomenon explored in the present study, particularly in the Swedish context, an inductive approach was implemented in order to describe women’s own experiences of perinatal clinical encounters, when living as undocumented migrants in Sweden.

Amongst the many qualitative methodologies considered, qualitative content analysis was selected. Methodologically, the approach is deemed appropriate when limited knowledge of the phenomenon explored exists, due to its inherent inductive nature accenting the importance of remaining open and impartial in the effort to describe and understand all facets and elements involved [40]. Building on Habermas’ [44] theory of communicative action, Prior [45] contended that, whilst early epistemological origins of content analysis revolve around the analysis of communication, its underpinnings acknowledge the importance of meaning in words and exchanges. As Prior [45] astutely noted, communicative action “rests at the very base of the lifeworld, and one very important way of coming to grips with that world is to study the content of what people say” (p. 360).

Furthermore, in its constructivist form, the use of qualitative content analysis in healthcare settings builds on the accumulation of meaning-units observed in patient and/or practitioner narratives, and thus allows for insight surrounding tangible issues that might otherwise go unseen [40, 45].

### Participants

The sample included undocumented migrants and EU citizens without residency permits who were pregnant and/or had given birth in Sweden. Recruitment was handled by multiple parties; two nurses and two of the authors of the present study at NGOs providing healthcare for undocumented migrants; a midwife at the ANHC in association with women’s perinatal care appointments; and a cultural doula from an NGO providing women supportive doulas during pregnancy and childbirth. Fourteen women were approached, and of these, one declined participation. The sample thus consisted of 13 women, ranging from 18 to 36 years of age and living as undocumented migrants, stemming from Macedonia, Romania, Bosnia, Albania, Somalia, Afghanistan, Serbia, Chechnya, Morocco, and Kosovo (see Table 1, on page 10, for further demographic details).

**Table 1:** Participant demographics

ID	Parity	First ANHC visit (GW)	Dating of pregnancy	Prenatal interview (GW)	Postnatal interview (months PP)	Legal status
1	2-gravida, 0-para	GW 10	Yes	28	11 months	Asylum claim rejected
2	1-gravida, 0-para	GW 16–20	No	35		Unregistered
3	2-gravida, 1-para	GW 6	Yes		2 months	Visa expired
4	2-gravida, 0-para	GW 18	Yes	30	3 months	Asylum claim rejected
5	1-gravida, 1-para	GW 17	Yes		2 months	Asylum claim rejected
6	3-gravida, 2-para	GW 6	Yes	36	2 months	Asylum claim rejected
7	5-gravida, 5-para		No		2 months	Unregistered
8	2-gravida, 1-para	GW 17–19	Yes	32		Visa expired
9	2-gravida, 1 para	GW 7	Yes	33	2 months	Asylum claim rejected
10	2-gravida, 1-para	GW 24	Yes	32		Asylum claim rejected
11	1-gravida, 0-para	GW 4	Yes	40	2 months	Asylum claim rejected
12	2-gravida, 2-para	GW 18	Yes		2 months	Unregistered
13	1-gravida, 1-para	GW 7	Yes		1 month	Asylum claim rejected

Participants stemmed from 10 different countries, and had experienced clinical encounters in Sweden during pregnancy and childbirth. The average participant age was 26

Eight participants were interviewed during their pregnancy, and follow-up interviews post-partum (PP) were carried out with five. Another five were only interviewed PP. Attrition in PP follow-up interviews was due to multiple factors: three declined to partake in further interviewing; one was unexpectedly forced to leave Sweden (attempts to reach her abroad were unsuccessful); one could not be interviewed due to her geographical location; and one woman failed to attend her planned interview. Thus, the data included in the present study’s analysis was gathered across 18 interviews.

### Data collection

The interviews took place in tandem with women’s visits at NGOs, or at a location of their choice, and one interview was conducted via Skype. Initially, the interviews were performed by two researchers (MB and JR) in tandem, with the purpose of gaining synthesis in interviewing technique and style, before proceeding to interviewing participants alone [41].

The interviews were unstructured in nature, and initiated with the open question: *What is your experience of healthcare encounters during pregnancy and childbirth, and what are your experiences of being pregnant and giving birth when living as an undocumented migrant?* Follow-up questions and probes were then guided by what each participant chose to discuss, in order to allow the women to influence the direction of their narratives, and facilitating the interviewer to cover a wider range of the phenomenon explored [41, 42, 46]. As the interviews came to a close, demographic characteristics were recorded (see Table 1), and debriefing ensued.

Language barriers existed for eight of the women, and thus the presence of interpreters was offered and organized for their interviews. The participant’s own preference in these cases were heeded, and this resulted in a professional interpreter being used in one case, a cultural doula in another, a volunteer interpreter in a third, and adult relatives in the remaining five.

The interviews lasted between 14 to 75 min (the average duration was 36 min, resulting in a total of 648 min of data collected). All were audio-recorded, with the exception of two. In these cases, as participants felt uncomfortable with the concept of recording their narratives, interview notes were taken, and later transliterated. All audio-recordings were transcribed verbatim, and cross checked by both interviewers to ensure accuracy and consistency prior to data analysis [42, 47].

### Data analysis

Initial steps of the data analysis process involved reduction and sorting, in order to exclude data not relevant to the explored phenomenon [40, 48]. The data was then independently analysed using qualitative content analysis, in accordance with Graneheim and Lundman’s [40] methods. This entailed identifying meaning-units relevant to the study’s aim and objective, with the purpose of constructing a composite description of the participants’ lived experiences of the explored phenomenon [48, 49].

Miles, Huberman, and Saldaña’s [49] theory of immersion was also implemented throughout the data analysis phase, by ensuring that the researchers involved in analysis were highly familiar with all the data prior to initiating open coding, in order to maintain an overarching understanding of key emerging patterns and concepts. Furthermore, all transcripts were revisited repeatedly by the research team, in the effort to obtain a concentrated perspective [50]. The emergent codes were then sorted into subcategories, which in turn subsequently merged into the surfacing main categories identified. This process allowed for the identification of major overarching themes that depicted the phenomenon explored, along with the key elements of interest.

Selected quotations from the interviews (with minor grammatical adjustments) have been included in the results section, in order to provide readers with the opportunity to evaluate the interpretations and credibility of the analysis conducted [42, 49].

### Ethical considerations

Ethical approval was obtained from the Gothenburg Regional Ethics Committee (Ref: 429–15). This study, in its entirety, was carried out in line with the Declaration of Helsinki [51]. All women were offered both verbal and written information about the study, and all were explicitly informed of its voluntary nature. They were also assured that the data collected would be treated confidentially, and were informed that they were free to withdraw at any time, without reason, and that withdrawal would not affect their access to healthcare. They were also informed that the interviewers were not connected in any way to the Swedish authorities. Debriefing occurred following each interview. A deep ethical consideration in regard to the sensitive and vulnerable position participants were in was maintained throughout [42], and in cases where a history of negative experiences were disclosed during the course of an interview, follow-up contact was offered. One participant was also assisted in booking an appointment with a postpartum psychologist after her interview, and another was referred to a psychotherapist at an NGO for further psychological appraisal.

## Results

Upon analyzing the data collected, two major themes emerged: (1) Experiencing acute distress when suffering subpar and neglectful clinical encounters (see Table 2, on page 43, for a full overview of all relating categories and subcategories); (2) Feeling acknowledged and empowered through experiencing positive clinical encounters (see Table 3, on page 44, for a full overview of all relating categories and subcategories).

**Table 2:** Theme 1. Women living as undocumented migrants experience acute distress when suffering subpar and neglectful treatment in clinical encounters

Sub-categories	Categories	Theme
Having prescriptions being filled out incorrectly Healthcare staff questioning women’s right to services Being prepared for having to argue for their rights to Healthcare access Fearing misinformed staff would contact authorities Having experienced being denied care, including emergency care Having to pay in full due to lacking documentation or later receiving fee invoices Encountering uniformed healthcare staff diminished help-seeking behaviors and caused distress	Experiencing deficits in healthcare personnel’s awareness and knowledge	
Observing healthcare personnel’s behaviors closely to assess intentions Feeling ignored, devalued, and questioned Fearing being treated inadequately and unequally Not receiving care and information induced acute distress Healthcare professionals normalized women’s concerns, and did not take concerns seriously Fearing being assessed as parents and reported Distrusting medical competence Perceiving intolerance/inflexibility in healthcare staff Feeling healthcare professionals were uninterested	Suspecting adverse intentions and perceiving neglectful behavior in healthcare professionals	Experiencing acute distress when suffering subpar and neglectful clinical encounters
Being informed of potentially adverse pregnancy outcomes induced acute panic Healthcare staff’s inability to communicate risks involved in complications caused confusion Having feelings of ambiguous and guild surrounding abilities as mothers when being informed of adverse outcomes Feeling an extreme lack of control in their circumstance and in their ability to convey their needs Fears of being deceived induced further distrust Fearing being misinterpreted by professional interprets	The need for functional communication with healthcare personnel and interpreters	

The table displays one of two overarching themes, along with its relating categories and sub-categories, and describes women’s experiences of clinical encounters in perinatal care as undocumented migrants

**Table 3:** Theme 2. Women living as undocumented migrants feel acknowledged and empowered through positive clinical encounters

Sub-categories	Categories	Themes
Wanting to be treated like all pregnant women Needing a sense of safety and security Feelings of trust decreased distress and fear Feeling grateful and joyous when healthcare professionals were welcoming, empathic, and caring Feeling safe when perceiving that respect and equal treatment were provided Appreciating healthcare professionals who were approachable and more personal Positive healthcare behaviors influenced attendance Being able to ask healthcare staff questions beyond during visits increased trust Joyous healthcare environments promoted happiness Feeling that being acknowledge increased wellbeing	Displays of trustworthiness in healthcare professionals conveyed safety and security	
Feeling gratitude for being provided adequate support Feeling thrilled when staff spoke their language Valuing information on how to handle childbirth, the postnatal phase, and impending motherhood When basic needs were met experiences were positive Appreciating perceptive healthcare professionals Being taken seriously during emergency situations increased trust in medical competence	The need for informed and flexible perinatal care	Feeling acknowledged and empowered through positive clinical encounters
Finding it difficult to discuss their concerns and fears Worrying about protecting and providing for their unborn child and pre-existing children Sharing longing for the baby’s arrival induced positive feelings Experiencing nesting behaviors, joy, and anticipation Developing coping strategies and striving for meaning in life	The need for positive reinforcement in coping with pregnancy and motherhood	
Unawareness of rights led to delaying seeking antenatal care NGOs educated women on their right to healthcare access NGOs/social networks empowered women to claim their rights NGOs/social networks facilitated positive experiences Feeling safe in sharing pregnancy with a supportive partner	Empowered by NGO’s and social networks in seeking healthcare	

The table displays one of two overarching themes, along with its relating categories and sub-categories, and describes women’s experiences of clinical encounters in perinatal care as undocumented migrants

Experiencing subpar treatment and neglect due to perceived adverse intentions, and/or deficient knowledge and awareness amongst healthcare personnel resulted in the women feeling discriminated against. Such experiences also appeared to cultivate extreme levels of anxiety and fear surrounding utilizing healthcare services. Contrastingly, the participants who took part in this study reported feeling safe and secure when encountering welcoming healthcare professionals who made efforts to provide them with adequate support. This in turn diminished concerns and cautiousness relating to seeking care.

Throughout the themes, categories, and subcategories observed, the women’s daily lives appeared impacted by acute levels of stress and uncertainty. Previous traumatic experiences, such as having witnessed violence, having been a victim of violence, or having felt the threat of violence, were reported to be severe sources of stress and anxiety during both pregnancy and childbirth. Having lived in threatening environments was described as negatively impacting one’s mental health (which displayed itself in the form of self-reported insomnia, cognitive issues, and depressive symptoms), and feelings of being acutely exposed and unsafe during pregnancy were repeatedly raised*.* Going through pregnancy without their partners, not knowing where the partner was located (or safe) further enhanced these emotions.

The aforementioned factors, collated with the extreme loss of control associated with living as an undocumented migrant, were described as leading to an overall loss of energy. This was further aggravated by fears of being discovered and deported by the Swedish authorities. Intentional efforts to avoid attracting attention led to isolation, and concerns in regard to how this affected their entire family (particularly pre-existing children) were prevalent.

### Experiencing acute distress when suffering subpar and neglectful clinical encounters

#### Experiencing deficits in healthcare personnel’s awareness and knowledge

Reports of lacking knowledge amongst healthcare professionals surrounding how to approach and relate to undocumented migrant patients were present throughout the data. Insufficient knowledge displayed itself in a variety of ways, such as the reoccurrence of questioning the women’s right to healthcare services, and being unaware that this population encounters difficulties when collecting prescription medication from the pharmacy. Ultimately, this deficiency was described as developing substantial obstacles in seeking care.

Participants who were knowledgeable about their legal rights reported being consistently prepared for having to argue for their right to healthcare access when encountering healthcare personnel.

Mistakes that could occur due to misinformed or misconceived healthcare personnel led to the development of fear amongst the women, largely related to concerns that clinical encounters could consequentially result in the authorities becoming aware of their location – and thus, leading to their deportation. This was described as follows:

*“We were somewhere to take blood tests. They did not know how to deal with us. We said we were undocumented… Hidden, and she said that my Swedish Reception of Asylum Seekers Card was very old, so it couldn’t be accepted. I said that we were undocumented, so we have nothing to do with the Migration Board… But she didn’t understand, she said the card was old and that I have to have a valid one, so that is why she couldn’t trust it, and that maybe I am hidden and so on. They do not know what to do… With the papers to register. Someone else also came, and she didn’t know either. But we didn’t know what they would do… It is always unsettling… They might write and send something to the Migration Board. I am absolutely very worried about that.”* (I.1).

Many women reported experiences of having been denied emergency care, specialist healthcare, and antenatal care. Several had also been withheld access to free transportation to the ANHC when care was needed, as well as equipment needed following childbirth (such as breast pumps). Multiple cases where healthcare professionals professed doubt in regard to the women’s undocumented status were also reported. This could result in registering the women as fully paying patients, due to the absence of documents clarifying their identity. Most women were wrongly informed that they would have to be charged for visits at the antenatal care unit, and invoices were sent to several of them following the delivery of their babies.

The aforementioned experiences resulted in high degrees of distress, and led to the development of fear, both for the women’s own safety, and for the lives of their babies. Experiences of having been refused healthcare induced feelings of worthlessness and grief, often leading to a general distrust in healthcare professionals. Due to this lack of faith in the healthcare system, several women reported cancelling pregnancy check-ups, and/or refraining from attending their appointments. Feelings of uncertainty, anxiousness, and distress were compounded by the fact that many felt they did not have anywhere to turn with their grievances. One women recounted:

“*I sought care for severe pains, I had waited from twelve in the day to twelve at night. We did not receive any examination. They wanted four thousand crowns* (equivalent to approximately 380 GBP) *for conducting the examination and they did not even touch one’s stomach, nothing... They took us in on a referral. They came back and said that ‘we can do nothing because you do not have any papers, you are undocumented’, after having sat there for 10 hours… We felt ignored and drove home.”* (I.2).

#### Suspecting adverse intentions and perceiving neglectful behavior in healthcare professionals.

Attitudes of healthcare professionals appeared significant, both in regard to whether care was provided, but also in relation to the ways in which it was carried out. Both verbal and non-verbal displays by healthcare professionals were appraised, and reported as crucial in the women’s own assessment of whether personnel’s intentions were malignant or benign. Many participants described experiencing subpar treatment, such as not being given access to care or not receiving qualified care. Reports of having experienced feeling ignored and questioned in healthcare settings were abundant. How the women were approached was depicted as inducing fears that healthcare professionals would treat them inadequately, or that assistance would be neglected.

In cases where women had been denied access to care, they felt that their needs and reasons for seeking healthcare were not taken seriously. When healthcare professionals failed to answer questions, or abstained from performing examinations, this resulted in the development of further anxiety, stress, and resistance towards visiting healthcare establishments. Healthcare professionals were also perceived as normalizing what the women themselves experienced as complicated conditions. This was noted in an account from a woman who had sought emergency care for premature rupture of membranes:

“*They contended that it was not amniotic fluid, but rather that I probably had urinated on myself. I stated that I had already birthed four children and that I know the difference between urine and amniotic fluid… They asked if I had tuberculosis in my family and she said I had to do a tuberculosis test. And I said we have no history of tuberculosis in my family, but they took the test anyway. But they never looked at the amniotic fluid and never took a cardiotocography… During the following week fluid and blood kept leaking. I was encouraged daily to go to the hospital, which I did not dare do. I did not feel it was worth it as they did not believe me anyway. Finally, I took myself there and they took tests and saw that there was no amniotic fluid left. Then they almost scorned me because I had not come in sooner. I tried to explain that I had been there earlier...”* (I.7).

The women reported distrust and fear regard to the Swedish authorities. This was especially expressed amongst EU citizens, as they had heard that social services could place their babies in foster care if they were deemed unfit as parents. Doubts concerning healthcare professionals’ medical competence were also highlighted, for example, in the form of distrust as to whether a vaginal or surgical delivery would be medically preferable. Many participants had experienced neglectful treatment, which resulted in developing high levels of sadness and fear. Participants reported feeling unheard in clinical encounters, inducing further anxiousness. When healthcare professionals failed to satiate the women’s needs, feelings of grief and resentment appeared to emerge:

“*No one listened to my wishes. I was forced to give birth vaginally, regardless of the pre-existing risks I had. Just like a mechanic knows a car, I know my body. I knew it would not work. They did not listen to me.”* (I.9).

When lacking tolerance on part of healthcare professionals was perceived, feelings of insecurity and anxiousness augmented. Failures to display flexibility toward the women’s needs and requests (for example, being denied a female physician in spite of holding strong religious beliefs) resulted in feelings of discrimination. It was also reported that some healthcare professionals demonstrated poor interest, or appeared deficient in their will to provide care. As one participant reported:

“*We got a midwife that we experienced as racist so it did not feel right and so, we requested to get a new one… it was like she did not care much about us… she wanted us to go back to our home country and she barely looked at us. We did not exist. Also, it was very hard, she pressed hard on my stomach… And there was another couple who had been to her and they got sent back to their home country. We got worried that she did not want to help us… got afraid that she would hurt the child. We arrived a little late, she got extremely angry at us and told us off. She said ‘why did you come, we have other things to do’.”* (I.2).

The women reported feeling less valued when healthcare professionals made distinctions between them and women of Swedish origin, and avidly reported sensing that they had not been provided equal treatment. One woman described her experience as follows:

*“I had a catheter and was in a lot of pain. I could barely reach out for my pain medication. When I said that I was hungry no one came with food. I barely had the energy to feed the baby.”* (I.9).

#### The need for functional communication with healthcare personnel and interpreters

Accounts of having suffered complications such as preterm births, preeclampsia, SGA-babies, stillbirth, and postpartum bleeding, surfaced during the interviews. Several women described that clinical encounters which revealed complications or potentially adverse outcomes during their pregnancies created severe distress. Finding themselves faced with having to make difficult decisions, in a foreign country without a social network, fueled acute panic. Confusion surrounding information received relating to arising medical conditions was voiced, particularly when healthcare professionals were unable to adequately explain the risks involved for both mother and child. The women also reported that the information provided induced a sense of disbelief in their own abilities, and led to the fostering of ambiguity, guilt, and self-criticism in their role as mothers/mothers to be.

Not being able to communicate or understand necessary information provided by healthcare professionals often resulted in feelings of losing control of what was happening to them – in an already acutely uncontrollable situation. Similar feelings were voiced in relation to being misunderstood, and not being able to adequately convey one’s needs. One participant reported the following:

*“I rang the bell on several occasions as I wanted help, I was worried that something was wrong with the baby which was just screaming and screaming. After a long while staff entered and said something incomprehensible in Swedish and then just left again. I was hoping that she was going to come back again with an interpreter. That never happened. This happened on several occasions...”* (I.11).

A sense of being deceived, or of not being accurately informed on conversations concerning their health and position further fueled feelings of distrust and insecurity. This was compounded by the presence of distrust toward interpreters hired by healthcare establishments. These fears stemmed from pre-existing cultural and geographical frictions and conflicts, and resulted in experiences of distress and discomfort when sharing personal details and problems for the interpreters to translate. This issue was apparent throughout the interviews. Due to this absence of interpreters the women felt they could trust, relatives were often pushed to take on the role of interpreter in emergency situations, which could also lead to personal discomfort.

### Feeling acknowledged and empowered through positive clinical encounters

#### Displays of trustworthiness in healthcare professionals conveyed safety and security

When considering the individual needs of the women who partook in this study, many were in large part similar to those of all expectant mothers. Overall, the women described a need for a sense of trust and safety when seeking perinatal care. Feelings of gratefulness and joy were reported to increase, and anxiety and fears to decrease, when encountering healthcare professionals who made active efforts to establish trusting clinical relationships. Such relationships appeared to be cultivated through being welcoming, and displaying empathic concern toward both the women and their unborn babies. When the women experienced being given treatment equal to that of all pregnant women, by being met with respect and humane commitment, it resulted in them feeling safer.

Participants reported that healthcare professionals who were more personal in their approach enabled them to share their thoughts and feelings. Such exchanges provided the women with energy-enhancing experiences. One participant’s description of such an occurrence read:

*“She looks after me attentively, and it doesn’t look like a doctor-patient relationship… Because she helped me one time, I didn’t have anything to eat. She gave me fifty Swedish crowns* [equivalent to approximately 4.50 GBP]*. I started crying because it was a very good gesture on her part.”* (I.4).

The women contended that the way in which healthcare professionals treated them had a significant impact on their appointment attendances. They also reported hat when staff were available to answer questions, beyond during regular visits at the ANHC, they felt an increased level of trust. This also assisted in creating an open relationship with healthcare professionals, where the women felt free to communicate their anxiety and fears.

Atmospheres of joy and laughter in healthcare settings made the women feel happy, and increased their wellbeing. This was also argued to be true in the delivery ward, as to be received with welcoming attitudes made them feel acknowledged, and promoted satisfaction following deliveries. The importance of healthcare professionals being present and supportive in the delivery room was raised as facilitating the management of labor pains. One woman articulated this as follows:

“*The best thing the midwife did for me was to sit by the bed, at eye-level, hold my hand, and acknowledge me. That was the best in order for me to feel secure as a woman – that I was heard.”* (I. 12).

### The need for informed and flexible perinatal care

The interviewees expressed amazement and gratitude toward healthcare professionals who provided them with adequate support, for example, by referring the women to specialist care for physical/psychological appraisal, or offering an interpreter. In cases where midwives who spoke the women’s native language were encountered, this was highly appreciated.

Several participants reported feeling poorly prepared for childbirth, and this increased their anxiety levels. Adequate support and information in regard to handling early phases of labor and delivery pains was desired, and the value of knowing relaxation exercises was acknowledged. Adequate support was described as having been provided information, receiving desired pain relief, and being assisted in remobilizing following delivery. Postnatal care was experienced as positive when basic needs were met, such as nutrition and basic intimate hygiene. It was also appreciated when healthcare professionals were perceptive and proactive, for example, by noticing how exhausted the women were and offering them assistance. Furthermore, some women voiced a need for support with breastfeeding, and/or in learning how to tend to their newborn. Opportunities to speak with other women who shared in their circumstances were also voiced as holding value.

In emergency situations during pregnancy (for example, in the case of premature contractions), several women experienced having been taken seriously, and receiving adequate support and information from healthcare professionals. These women described that being in an acute situation gave rise to a strong sense of fear and distress, and that such feelings were easier to bear when they felt acknowledged and heard in clinical encounters. Being given the opportunity to share their concerns, grief, and anxiousness with healthcare professionals in complex situations provided comfort.

When action was taken rapidly and efficiently, the women’s experiences of healthcare provision was positive, and perceived as supportive. Healthcare professionals’ medical competence was perceived as highly trustworthy in emergency situations where adequate efforts were made to prevent complications, for both the women and their unborn babies. This was depicted by one participant as follows:

“*The ambulance came really fast. Five minutes past and the ambulance arrived, and when we got to the hospital, they took care of… as in, they were not slow… no, they knew what they had to do… They all tried to do their best to ensure that the child remained in the belly.”* (I.13).

#### The need for positive reinforcement in coping with pregnancy and motherhood

Although acknowledgement in clinical encounters was reported to enhance the women’s well-being, difficulties in discussing anxiety and fears remained persistent even in cases where empathic concern was conveyed. Overall, an avoidance in relation to discussing their own circumstances with healthcare professionals was present, even when direct questions were posed. Many women described a sense of loneliness, of being exposed and vulnerable, and feeling unable to share their plight. This was articulated by one participant as follows:

“*My midwife always asks me how I feel, but I’m a person who has a very difficult time talking about how I feel. I can only do this in moments when I feel very, very bad.…. I used to be independent… I feel bad when I ask for help from other people. I just don’t want to…”* (I. 4).

Social vulnerability was reiterated as being enhanced during pregnancy, particularly for women with pre-existing children. The threat of being evicted, being homeless, having to move frequently to new settlements, and lacking economic stability resulted in increased levels of anxiety. This in turn exacerbated worries in regard to meeting the basic needs of their unborn baby and pre-existing children. Concerns surrounding the protection of their own lives were surpassed by the fear of not being able to provide their offspring with a safe childhood. This was described by one woman as follows:

*“I felt very depressed following the delivery. No one listened to me. I live for my children, if something were to happen to them I don’t want to live. Life is hard. I can’t afford food for the children. I have been prescribed medication but if I take them, I can’t breastfeed, and then my son has to go hungry because I cannot afford to buy food for him. The worst is that I cannot afford food.”* (I.9).

Notwithstanding this, being given the opportunity to share their longing for their unborn baby with others was described as a joyful experience. Preparing for the baby’s arrival was reported as inducing positive feelings. Throughout the data, it was clear that these pregnant women were like most other women, and that their undocumented status was not a part of their personality and identity as human beings, but rather a predicament they found themselves in due to the circumstances they suffered in life. Nesting behaviors, anticipation, and concerns for their offspring mimicked that of many pregnant women seeking antenatal care, and the joys and highlights surrounding this were easily recognizable in their narratives:

*“We bought a bed for her… and then I was so happy…”* (I.2).

The women expressed having developed strategies to cope with the worries of everyday life, such as trying to focus on happiness, trying to focus on one day at the time, and trying to focus on the future rather than the past. This was illustrated by the following interviewee:

*“If it hadn’t been for our children’s sake, then we would have returned to our home country. But we believe and hope that Sweden is a better place for our children. Here we will not be bombed… It is my children that give me what it takes to manage every day, as my husband and I fight on for their sakes. So that they can one day get a safe and real life.”* (I.6).

A desire for meaning in life, with a future where their families could reside in security, was repeatedly voiced. However, for many, hopes and dreams were suspended whilst awaiting approval of their asylum applications and appeals. A peak aspiration was to receive a residency permit. Nevertheless, women expressed that life as an undocumented migrant in Sweden gave them freedom when compared to their previous lives. Escaping from their home countries meant being able to have aspirations for a better future, and to live in freedom and protection:

*“…I am in Sweden and free. I can do what I want…”* (I.13).

#### Empowered by NGOs and social networks in seeking healthcare services

Overall, the participants reiterated the immense importance of engagement and support from NGOs, religious communities, and cultural doulas. Lacking knowledge surrounding their rights to healthcare appeared as the reason for the women seeking perinatal care only in the final phases of pregnancy, and NGOs were reported as holding a significant role in educating them on their rights in this regard. Being informed of their rights encouraged and strengthened the women to visit the ANHC, the hospital, and emergency care. For those who had previously suffered negative experiences when encountering healthcare professionals, this support was of elevated importance.

For many, economic support from NGOs, such as the provision of bus fare to the ANHC, was essential in facilitating women in attending their booked antenatal care appointments. The provision of volunteer cultural doulas capable of speaking the women’s native language was of immense value, and provided support that extended beyond childbirth. To receive support from a doula with whom they could easily communicate reinforced positive experiences in relation to labor and delivery. Participants also described the value of having someone to advocate for them in complicated situations:

*“She told me that, because she didn’t know how long I had been here, and that I’m hiding, I had to pay because she thought I was here like a tourist… Then after having explained the situation to her, she told me again that she didn’t know. So, I had to come here* [to the NGO] *to tell them, so that they could call* [the healthcare establishment]*. I was scared, not for myself, but because I didn’t know what would happen to my baby. Who will check on me, and where will I go.”* (I.3).

Becoming aware of their own legal rights gave the women strength to request qualified care for themselves and their newborn babies, and to object to improper treatment in clinical encounters. Nevertheless, many reported that familiarizing themselves with the healthcare system’s structure and processes was problematic. Social networks, along with relatives who were familiar with the Swedish healthcare system, were argued to be of vital importance, as they assisted in booking consultations and interpreters at the ANHC. At times, they also interpreted for the women, or helped by driving them to their appointments. This support was deemed essential in order to master some of the predicaments involved with being an undocumented migrant, as ultimately, it facilitated the utilization of much needed healthcare services. Finally, having a supportive partner, someone comforting to share the burden of grief and hope with, was voiced as an important component in feeling safe and secure during pregnancy.

## Discussion

The present study aimed to describe women’s experiences of clinical encounters during pregnancy and childbirth, when living as undocumented migrants in Sweden. Findings indicated that women who felt heard and acknowledged by healthcare professionals were strengthened and encouraged by the encounters. Being met with welcoming engagement and empathic concern resulted in increased trust in healthcare personnel, and thus made the women feel more secure about seeking care.

The women’s narratives surrounding clinical encounters, and the need for encouragement and reinforcement shared strong similarities to those illustrated in Robertson’s [35] findings on migrant women’s experiences of childbearing in Sweden. However, an additional dimension surfaced in the present study’s data, which was the presence of a constant underlying anxiousness and fear of not being able to protect their newborn child and pre-existing children, and of being discovered and deported. This difference is likely related to the undocumented status of this study’s sample, as it was clear throughout the data that the uncertainty in their futures led to acute levels of anxiety.

Overall, the analysis illustrated a prevalence of vulnerability and distress in the recruited sample. The prominent themes that emerged through data analysis depicted the plight of women living as undocumented migrants as stress and anxiety-laden. This magnified the women’s need for a sense of safety, security, and trust in encounters with healthcare professionals, throughout pregnancy, childbirth, and in the early phases of motherhood. When experiencing sup-par or neglectful treatment, the women perceived healthcare professionals as holding adverse intentions. This led to a sense of distrust, and resulted in an overall reluctance towards seeking healthcare services. When encountering healthcare professionals who were supportive and present, and who showed empathic concern, the women felt enabled to communicate difficult issues, allowing them to share their feelings of distress. The findings of the present study therefore come with the implication that healthcare professionals have an ethical responsibility to appear welcoming and encouraging in clinical encounters with women living as undocumented migrants. Moreover, additional efforts should be dedicated towards promoting self-esteem and empowerment, in order to assist these women in managing their extremely vulnerable situation during pregnancy, childbirth, and the early phases of motherhood.

As building trustworthy clinical relationships in perinatal care is reliant on women attending antenatal care, healthcare professionals must remain sensitive to impeding factors for women living as undocumented migrants noted in this study. Without interpersonal efforts that rely on being informed and knowledgeable surrounding these women’s needs, positive clinical relationships cannot be established. This is paramount, as issues such as failing to acknowledge the women’s presence, appearing dismissive, or erroneously charging fees can result in the cultivation of fears surrounding seeking potentially vital healthcare services, and thus it is a matter of patient safety. Explicitly addressing these problems in healthcare settings is of increased importance, as their prevalence is also supported by Kvamme and Ytrehus’s [25] study, which highlighted that healthcare fees, fears of being reported to authorities, poor language skills, and financial difficulties resulted in delaying seeking needed healthcare services.

The analysis further illustrates that women living as undocumented migrants are an acutely vulnerable and exposed group in society, in need of additional support in perinatal care. Several women in this study’s sample reported having suffered complications, such as preterm delivery, preeclampsia, SGA babies, stillbirth, and postpartum bleeding, and these are also issues highlighted throughout the literature as prevalent amongst migrant women [28, 32]. In Feldman’s [34] study, midwives reported experiencing ethical dilemmas when they were unable to provide asylum-seeking women with the needed continuity of care. Kvamme and Ytrehus [25] found that such dilemmas also involved feeling stifled by not being able to provide migrant women with aid beyond the necessary medical assistance, even though this would be direly needed. Nilsson and Saxäng’s [39] findings illustrated how midwives caring for asylum-seeking women experienced antenatal care to be subordinate considering the distressful living circumstances appertaining to this population, and thus recommended allowances be made for extended appointments in these cases. The need for continuity of care, additional assistance, and extended time for developing trusting clinical relationships were all supported by the findings of the present study. This accents the importance of policy-driven organizational facilitation that ensures healthcare professionals are provided with adequate time and knowledge needed in providing women living as undocumented migrants with flexible treatment. Throughout this study’s data, it was apparent that the need for such efforts is vital in promoting the development of trusting, safe, and secure clinical relationships with these women. This is all the more important when considering that this group of women suffer high degrees of social vulnerability, and psychological traumatization.

Another major finding of the present study was the indication that women living as undocumented migrants could be denied their legal right to healthcare access. Moreover, in cases where healthcare was provided, these women were at risk of suffering subpar treatment and neglect at the hands of healthcare professionals. Being denied access or suffering negative experiences in healthcare settings led to the cultivation of fears of being personally harmed, or of exposing their unborn baby to harm. Ultimately, this resulted in the women refraining from seeking healthcare services, or cancelling booked appointments, increasing the risk of aggravating their conditions. In 2015, the Swedish Agency for Public Management [18] followed-up on the implementation of legislation intended to expanded access to healthcare for undocumented migrants (Act 2013:408), and reported that the most common obstacles amongst healthcare professionals for denying undocumented migrants healthcare related to lacking knowledge on various levels. This deficit in healthcare personnel’s knowledge surrounding the law was highlighted throughout this study’s data. Whilst women living as undocumented migrants in Sweden are legally entitled to perinatal care [14], results showed that the sample suffered adverse treatment, which included at times being denied their legal right to healthcare access.

Findings also revealed that the women who were EU citizens, but remained in the country without legal status, appeared to experience more adverse treatment in clinical encounters than other women recruited in this study. However, due to the very limited rate of EU/ESS participants in the sample, further explorations on this specific population and their experiences of perinatal care and healthcare access in Sweden remains needed. Whilst the present study’s results support the Swedish Association of Local Authorities and Regions [52] statement that EU migrants lacking insurance cards should be paying in full for healthcare services [52], it conflicts with the National Board of Health and Welfare’s position on the issue [15]. It also remains grossly in conflict with human rights conventions [12] and ethical codes from throughout the medical professions, including those put forth by the WMA [10] and the ICM [11], which are founded on respect for human dignity and for women as persons with full human rights. Fundamentally, such ethical principles are based on non-discrimination, mutual respect, and equity.

Previous research, along with reports from NGOs, have shown that the fears of being discovered by authorities which exist amongst undocumented migrants, have resulted in them abstaining from attending healthcare appointments [19, 25]. This was also highly present in this study’s findings, and thus further highlights the responsibility healthcare policy-makers and leadership hold in ensuring that healthcare providers cultivate sufficient knowledge surrounding undocumented migrants’ legal rights to perinatal care and healthcare access. Awareness of the vulnerable position women living as undocumented migrants hold, as well as remaining informed on their needs and their rights is a necessity in facilitating their access to healthcare. Ultimately, along with training healthcare personnel in human rights, particularly the Right to Health [53], efforts in addressing these aspects could effectively reduce unnecessary suffering for both the women, and their children. Furthermore, it can reduce medical risks, and thus becomes a matter of patient safety and equity.

This study’s results also indicated that the sample, to a high degree, was dependent on NGOs and social networks, both in enabling their access to healthcare, and in promoting trust in healthcare professionals. Support from NGO’s, relatives, and cultural doulas were at critical points described as lifesaving. Being informed about one’s legal rights appeared empowering, and decreased fears surrounding seeking needed healthcare services. This supports findings from McLeish and Redshaw’s [54] study, which found that being supported by trained volunteers who remained outside the ‘social circle’ provided relief, promoted trust, rebuilt self-esteem, and increased empowerment – ultimately, facilitating recent migrant women to honestly share their feeling. Balaam et al.’s [38] findings also highlighted this phenomenon, as their study illustrated how volunteer workers assisted in identifying and providing alternative routes into the healthcare system, in order to ensure that migrant women receive the care they needed. Whilst previous findings referred to migrant women living as refugees and asylum-seekers, combined with the results of the present study it could be argued that healthcare organizations could improve their approach by learning from, and cooperating with NGOs who cater to migrant women, both registered and undocumented. Valuable lessons on providing support in a safe environment that truly address the biological, psychological, and social needs involved in pregnancy and childbirth when living in vulnerable circumstances could be shared.

Overall, the findings indicated a series of psychosocial, and physical consequences of enduring continuous stress and anxiety during pregnancy. The acute vulnerability in the women’s exposed living circumstances, along with fears of being discovered by authorities, appeared to result in both mental and physical distress. Exhaustion also played a role in the failure to attend booked healthcare appointments. Coping with traumatic memories and struggling with motherhood took its toll, and self-reported insomnia, depression, loss of energy, loss of concentration, loss of appetite, and constant high levels of stress and anxiety were prevalent throughout the interviews conducted. Previous findings list risk factors for PPD as stressful life events, migration, low socioeconomic status, and lack of social support [20], and migrant women have been found to be at increased risk compared to native-born women to develop the condition [17, 23, 30]. Whilst the sample recruited for the present study appeared to suffer from all of these risk factors, and whilst it is conceivable that risk factors amongst migrant women would be similar amongst migrant women without legal status, further research should explore risks relating to mental health during pregnancy and childbirth specifically amongst women living as undocumented migrants. Implications could be consequential in adequately training practitioners with the tools needed to provide informed psychological support during pregnancy, childbirth, and the post-partum phase.

The present study’s results highlighted an elevated presence of vulnerability, anxiety, and fears of being exposed to the authorities amongst the sample. These feelings were further compounded by difficulties in coping with motherhood. In spite of their distressing situation, the women made dedicated efforts to fulfil their children’s basic needs, even when this meant forfeiting their own. This dedication prioritization of children has also been highlighted by midwives caring for pregnant asylum-seeking women in previous findings [39]. In the present study’s results, fears of being discovered and deported by the authorities caused the women to worry extensively about their abilities to provide their children with a safe and normal childhood. These fears also led to social isolation, exacerbating their situations further.

Children raised in families with various social disadvantages are at increased risk of developing mental and physical ailments [55]. Children of psychologically traumatized parents who live in fear of being deported grow up in acutely distressing circumstances, where parents are often unable to provide their children the desired protection [55, 56]. It has also been found that maternal stress is associated with increased risks of acute illness within the infant’s first year of life [57], and both prior trauma and birth-related trauma have been associated with problematic childhood attachment [58]. The presence of these risks highlights the need for research focusing specifically on mothers living as undocumented migrants, in order to further explore how maternal stress and anxiety during pregnancy and the neonatal phase affects these children throughout childhood, adolescence, and into adulthood. Pending further empirical explorations, the findings of the present study depict a crucial need for adjusting perinatal care for women living as undocumented migrants to include a focus on the children (both expected and pre-existing), in order to meet specific needs stemming from their highly vulnerable and exposed circumstances. It has been argued that welcoming otherness, showing flexibility, and remaining sensitive to diversity, culture, and context are paramount in the provision of adequate and ethical mental health services [59, 60]. Encounters in perinatal care could serve as rich opportunities for practitioners to empower mothers living as undocumented migrants, through the promotion of healthy coping skills, and the provision of much-needed encouragement. Such efforts could ultimately positively affect outcomes for both the women, and their children.

### Strengths and limitations

The present study comes with several strengths and limitations. Major strengths revolve around the qualitative approach implemented in depicting the sample’s multifaceted narratives, as well as the focus on a population limited in their participation in research due to their often vulnerable and disadvantaged backgrounds. The recruited sample also depicted a broad range of personal characteristics, with the inclusion of diverse ages, parities, ethnicities, and educational levels [61]. Further strengths reside in the multidisciplinary research team involved in the study’s execution [50], as the authors hold backgrounds in medicine, midwifery, psychology, medical jurisprudence and ethics, healthcare science, and sociology, and additional guidance and constructive critique was sought from a social anthropologist and a phenomenologist.

Methodological considerations in regard to the risk of bias were also taken into account, and counteracted by involving the entire research team in reflexivity and quality control throughout the research process [50]. Reflexivity refers to the effort to become aware of and elucidate researchers’ preconceptions, often based on personal and professional experiences and own beliefs, in relation to the explored phenomenon [62]. Several of the authors held high levels of knowledge surrounding the phenomenon studied, reaped through backgrounds in relief work caring for the cohort in focus, and through medical and midwifery practice. During data analysis, awareness surrounding one’s own values, cultural background, and experiences were continuously reflected upon, in order to minimize the influence of these factors on the study’s result. It could also be argued that a certain level of pre-existing knowledge surrounding pregnant women living as undocumented migrants allowed for additional insight in promoting feelings of safety in participants. This in turn may have facilitated the women in sharing their narratives more openly.

To assess validity criteria, credibility was augmented by reflecting on deviating cases and accenting subtle differences in narratives, in order to maximize the authenticity of the analysis [40]. Transferability was ensured by providing a composite description of both data collection and the analysis process [43], and the study’s design could be implemented on similar populations in other areas. Whilst the sample was small, and primarily Eastern European, a certain degree of dependability exists, due to well-organized interviews, and the presence of prior multidisciplinary knowledge surrounding undocumented migrants and the studied context.

Several substantial limitations must also be acknowledged. Eight of the women spoke through interpreters. Whilst these interpreters primarily consisted of relatives or cultural doulas, depending on the participant’s preference, it could be argued that the women who partook in this study may have been impeded in expressing themselves freely. Although the interviewers maintained an astute awareness of this matter, and deemed that the women felt safe with their chosen interpreter, this limitation may have rendered the data collected limited in its range. It could also have resulted in the data suffering a deficit in more sensitive narratives, as well as those involving the women’s relatives themselves. Although all of the participants were offered interpreters, a professional interpreter was hired only on one occasion. Findings from data analysis indicated that this was likely due to reports of the women having previously suffered negative experiences when professional interpreters were present.

As participants reported fearing being denied care if they raised criticism, and as it was made known that the interviewers were midwifery students during the recruitment process, risks also exist that this may have affected the women’s willingness to describe on-going negative experiences they were being submitted to. Nevertheless, efforts were made to promote trust during interviews, ensuring the comfort of all participants. This was addressed in part by allowing a free choice of time and location for the interviews to take place, and by reassuring participants of their anonymity in reports of the study’s results [41, 42]. Power imbalances between the interviewers and the women were also considered [63], due to risks of promoting feelings of discomfort, vulnerability, and exposure during the interviewing process. This was addressed by allowing the women to choose the line of discussion, and ensuring to not probe further when distress was displayed. Ultimately, the level at which the women shared their concerns and vulnerabilities speaks to the level of participant-researcher trust achieved.

Finally, another major limitation stems from the fact that 11 participants were recruited through an NGO that at times caters to women in need of support in the event that they were denied healthcare. The prevalence of such experience may therefore be more present in the recruited sample. This warrants further research on broader samples receiving perinatal care, as well as those receiving no form of healthcare services at all, on national and international levels.

## Conclusion

The results of the present study have provided unique and potentially crucial insights surrounding women’s experiences of pregnancy, childbirth, and early motherhood, when living as an undocumented migrant in Sweden. The overarching findings indicated that the needs of undocumented migrant women were largely similar to those of all expectant mothers, but that due to their uncertain circumstances, flexible and informed care provision is essential. The data clearly illustrated how these women’s plights are accompanied by extreme levels of vulnerability, and that this affects all areas of life, including pregnancy and childbirth. Results indicated that how women living as undocumented migrants are treated in clinical encounters is highly salient, as whether positive or negative, these exchanges appear impactful.

The women who participated in this study described an acute need for developing trustworthy and secure relationships with healthcare professionals. Multiple experiences of poor and neglectful treatment, creating distrust and unwillingness to take part in antenatal care were reported. This led to a need for support from NGOs and social networks in enabling access to healthcare. Struggles to cope with traumatic memories, as well as feelings of guilt and ambiguity in relation to tackling motherhood whilst living in exposed conditions, compounded the women’s already vulnerable and distressing situations.

The present study contributes to the limited body of knowledge surrounding the plight of women living as undocumented migrants, by increasing understanding and awareness surrounding their experiences of enduring pregnancy and childbirth when submitted to such vulnerable and uncertain circumstances. The importance of meeting these women’s specific needs and concerns in perinatal care have also been raised, and presenting issues healthcare leadership and policy-makers should address have been highlighted.

Beyond the application of current legislation protecting undocumented migrants right to perinatal care, explicit efforts are needed in addressing the issues illustrated in this study’s findings. The promotion of an astute awareness amongst practitioners surrounding human rights and the Right to Health, along with a crucial need for dedicated adherence to ethical principles in clinical encounters appear paramount in improving the quality of care delivered. Ultimately, antenatal care could serve as a rich opportunity to identify women in need of further medical assistance, qualified psychological care, and/or psychosocial support during pregnancy in early motherhood, minimizing discrimination in healthcare access, and promoting patient safety and equity.

## Abbreviations

ANHC: Antenatal Healthcare Center
EU: European Union
GW: Gestational week
HIV: Human Immunodeficiency Virus
ICM: International Confederation of Midwives
NGO: Non-Governmental Organization
PP: Postpartum
PPD: Post-Partum Depression
PTSD: Post-Traumatic Stress Disorder
SGA: Small for Gestational Age
UN: United Nations
WHO: World Health Organization
WMA: World Medical Association

## References

[1] Cuadra CB (2012). Right of access to healthcare for undocumented migrants in EU: a comparative study of national policies. Eur J Pub Health.

[2] Castles S (2000). Ethnicity and globalization.

[3] State Public Reports. 2011:48: Vård efter behov och på lika villkor- en mänsklig rättighet [2011:48: Healthcare according to need and on equal terms – a human right]. Stockholm: Fritze; 2011. http://www.regeringen.se/rattsdokument/statens-offentliga-utredningar/2011/05/sou-201148/. Accessed 19 Mar 2018.

[4] Envall E, Vestin S, Björngren Cuadra C, Staaf A, Ascher H, Khosravi S, Socialstyrelsen (2010). Papperslösa [Undocumented migrants]. Social rapport [Social report].

[5] Swedish Migration Agency. Applications for asylum received 2000-2017. Migrationsverket; 2017. https://www.migrationsverket.se/english/about-the-migration-agency/facts-and-statistics-/statistics/overview-and-time-series.html. Accessed 22 Mar 2018.

[6] Vogel D. Size and development of irregular migration to the EU Clandestino research project - counting the uncountable: data and trends across Europe. European Commission & Citizens and Governance in a Knowledge-Based Society; 2009. http://irregular-migration.net/typo3_upload/groups/31/4.background_information/4.2.policy_briefs_en/comparativepolicybrief_sizeofirregularmigration_clandestino_nov09_2.pdf. Accessed 19 Mar 2018.

[7] United Nations Development Programme. Human development report 2009 - overcoming barriers: human mobility and development. New York; 2009. http://hdr.undp.org/sites/default/files/reports/269/hdr_2009_en_complete.pdf. Accessed 19 Mar 2018.

[8] World Health Organization. Constituition of the World Health Organisation. 2006. http://www.who.int/governance/eb/who_constitution_en.pdf. Accessed 19 Mar 2018.

[9] United Nations Human Rights Office of the High Commissioner. International covenant on economic, social and cultural rights (ICESCR), G.A. Res. 2200A (XXI), Art. xx. 1966.

[10] World Medical Association. Declaration of Geneva. 1948. https://www.wma.net/policies-post/wma-declaration-of-geneva. Accessed 19 Mar 2018.

[11] International Confederation of Midwives. International code of ethics for midwifes. 2014. https://internationalmidwives.org/icm-respurces/icm-publications/icm-core-documents.html. Accessed 19 Mar 2018.

[12] Hunt P. Report of the special rapporteur on the right of everyone to the enjoyment of the highest attainable standard of physical and mental health, − mission to Sweden: human rights documents. United Nations; 2007.

[13] The Rosengrenska Foundation. Information for patients - About the Rosengrenska Foundation. http://www.rosengrenska.org/information-for-patients. Accessed 19 Mar 2018.

[14] Ministry of Health and Social Affairs. SFS 2013:407 Lag (2013:407) om hälso- och sjukvård till vissa utlänningar som vistas i Sverige utan nödvändiga tillstånd [SFS 2013:407 Law (2013:407) regarding healthcare and treatment for certain foreigners staying in Sweden without necessary documentation]. Stockholm: Socialdepartementet; 2013.

[15] National Board of Health and Welfare. Vilken vård ska ett landsting erbjuda asylsökande och papperslösa? [What care should a county offer asylum-seekers and undocumented migrants]. 2013. http://www.socialstyrelsen.se/vardochomsorgforasylsokandemedflera/halso-ochsjukvardochtandvard/vilkenvardskaerbjudas. Accessed 19 Mar 2018.

[16] Swedish Association of Local Authorities and Regions. Hur länge kan EU-medborgare vistas i Sverige [How long can an EU-citizen stay in Sweden]. https://skl.se/ekonomijuridikstatistik/juridik/euratt/utsattaeumedborgare/eumedborgaresrattigheter/hurlangekaneumedborgarevistasisverige.7030.html. Accessed 19 Mar 2018.

[17] National Board of Health and Welfare. Vård för papperslösa. Vård som inte kan anstå, dokumentation och identifiering vid vård till personer som vistas i landet utan tillstånd [Healthcare for undocumented migrants. Care that cannot be defered, documentation and identification in care for people staying in the country without documentation]. 2014. https://www.socialstyrelsen.se/Lists/Artikelkatalog/Attachments/19381/2014-2-28.pdf. Accessed 19 Mar 2018.

[18] Swedish Agency for Public Management. Vård till papperslösa- En uppföljning av lagen om vård till personer som visats i Sverige utan tillstånd tillstånd [Healthcare for undocumented migrants – A follow-up of the law on healthcare for persons staying in Sweden without documentation]. 2015. http://www.statskontoret.se/upload/Publikationer/2015/201510.pdf. Accessed 19 Mar 2018.

[19] du Monde M (2016). Leagal report on access to healthcare in 17 countries.

[20] Collins CH, Zimmerman C, Howard LM (2011). Refugee, asylum seeker, immigrant women and postnatal depression: rates and risk factors. Archives of Women's Mental Health.

[21] Pusca A (2012). Roma in Europe: migration, education, representation.

[22] Ringold D. Roma and the transition in central and eastern Europe: the world bank; 2000.

[23] Munro K, Jarvis C, Munoz M, D'Souza V, Graves L (2013). Undocumented pregnant women: what does the literature tell us?. J Immigr Minor Health.

[24] Wolff H, Epiney M, Lourenco AP, Costanza MC, Delieutraz-Marchand J, Andreoli N, Dubuisson JB, Gaspoz JM, Irion O (2008). Undocumented migrants lack access to pregnancy care and prevention. BMC Public Health.

[25] Kvamme E, Ytrehus S. Barriers to health care access among undocumented migrant women in Norway. Society, Health & Vulnerability. 2015;6(1):28668. 10.3402/shv.v6.28668

[26] Wolff H, Stalder H, Epiney M, Walder A, Irion O, Morabia A (2005). Health care and illegality: a survey of undocumented pregnant immigrants in Geneva. Soc Sci Med.

[27] de Jonge A, Rijnders M, Agyemang C, van der Stouwe R, den Otter J, Van den Muijsenbergh ME, Buitendijk S (2011). Limited midwifery care for undocumented women in the Netherlands. J Psychosom Obstet Gynaecol.

[28] Almedia L, Caldas J, Ayres-de-Campos D, Salcedo-Barrientos D, Dias S (2013). Maternal healthcare in migrants: a systematic review. Maternal Child Health J.

[29] Small R, Gagnon A, Gissler M, Zeitlin J, Bennis M, Glazier R, Haelterman E, Martens G, McDermott S, Urquia M (2008). Somali women and their pregnancy outcomes postmigration: data from six receiving countries. BJOG : An International Journal of Obstetrics and Gynaecology.

[30] Villadsen SF, Mortensen LH, Andersen AMN (2009). Ethnic disparity in stillbirth and infant mortality in Denmark 1981–2003. J Epidemiol Community Health.

[31] Villadsen SF, Sievers E, Andersen A-MN, Arntzen A, Audard-Mariller M, Martens G, Ascher H, Hjern A (2010). Cross-country variation in stillbirth and neonatal mortality in offspring of Turkish migrants in northern Europe. Eur J Pub Health.

[32] Lu MC, Lin YG, Prietto NM, Garite TJ (2000). Elimination of public funding of prenatal care for undocumented immigrants in California: a cost/benefit analysis. Am J Obstet Gynecol.

[33] Wendland A, Ehmsen BK, Lenskjold V, Astrup BS, Mohr M, Williams CJ, Cowan SA (2016). Undocumented migrant women in Denmark have inadequate access to pregnancy screening and have a higher prevalence hepatitis B virus infection compared to documented migrants in Denmark: a prevalence study. BMC Public Health.

[34] Feldman R. When maternity doesn’t matter: dispersing pregnant women seeking asylum. Reproductive Health Matters. 2013;21(42):212–7.

[35] Robertson EK (2015). "to be taken seriously": Women's reflections on how migration and resettlement experiences influence their healthcare needs during childbearing in Sweden. Sexual & Reproductive Healthcare.

[36] Nkulu-Kalengayi FK, Hurtig AK, Ahlm C, Ahlberg BM (2012). “It is a challenge to do it the right way”: an interpretive description of caregivers’ experiences in caring for migrant patients in northern Sweden. BMC Health Serv Res.

[37] Nkulu Kalengayi FK, Hurtig A-K, Nordstrand A, Ahlm C, Ahlberg BM (2016). Perspectives and experiences of new migrants on health screening in Sweden. BMC Health Serv Res.

[38] Balaam M-C, Kingdon C, Thomson G, Finlayson K, Downe S (2016). ‘We make them feel special’: the experiences of voluntary sector workers supporting asylum seeking and refugee women during pregnancy and early motherhood. Midwifery.

[39] Nilsson K, Staxäng J. Caring for pregnant asylum seeking women – an interview study with midwives from a hermeneutic lifeworld approach. Gothenburg: Sahglrenska Academy; 2012.

[40] Graneheim UH, Lundman B (2004). Qualitative content analysis in nursing research: concepts, procedures and measures to achieve trustworthiness. Nurse Educ Today.

[41] Brinkmann S, Kvale S (2015). InterViews: learning the craft of qualitative research interviewing.

[42] Creswell JW (2013). Qualitative inquiry and research design: choosing among five approaches.

[43] Marshall C, Rossman GB (2016). Designing qualitative research.

[44] Habermas J (1984). The theory of communicative action.

[45] Prior L, Leavy P (2014). Content analysis. The Oxford handbook of wualitative research.

[46] Kvale S (2007). Doing interviews.

[47] Kowal S, O'Connell DC, Flick U (2014). Transcription as a crucial step of data analysis. The SAGE Handbook of Qualitative Data Analysis.

[48] Drisko J, Maschi T (2015). Content analysis.

[49] Miles MB, Huberman AM, Saldaña J (2014). Qualitative data analysis: a methods sourcebook.

[50] Cornish F, Gillespie A, Zittoun T, Flick U (2014). Collaborative analysis of qualitative data. The SAGE handbook of qualitative data analysis.

[51] World Medical Association. Declaration of Helsinki. https://www.wma.net/policies-post/wma-declaration-of-helsinki-ethical-principles-for-medical-research-involving-human-subjects/. Accessed 19 Mar 2018.

[52] Swedish Association of Local Authorities and Regions. Har utsatta EU-medborgare möjlighet att få hälso- och sjukvård i Sverige? [Do vulnerable EU citizens have access to healthcare in Sweden? https://skl.se/ekonomijuridikstatistik/juridik/euratt/utsattaeumedborgare/utsattaeumedborgarefragorochsvar.13958.html. Accessed 19 Mar 2018.

[53] International Organization for Migration. World migration report 2010 -The future of migration: Building capacities for change. 2010. https://publications.iom.int/system/files/pdf/wmr_2010_english.pdf. Accessed 19 Mar 2018.

[54] McLeish J, Redshaw M (2017). Mothers' accounts of the impact on emotional wellbeing of organised peer support in pregnancy and early parenthood: a qualitative study. BMC Pregnancy and Childbirth.

[55] Ascher H, Hjern A. Från apati till aktivitet. Teori och behandling av flyktingbarn med svår psykisk ohälsa [From apathy to activity. Theory and treatment of refugee children with severe mental illness]. Lund: Studentlitteratur; 2013.

[56] Ascher H. Refugee children living in hiding. What do we know and what don’t we know? Gothenburg: the Solstickan Foundation and Nordic school of Public Health; 2009.

[57] Phelan AL, Dibenedetto MR, Paul IM, Zhu J, Kjerulff KH (2015). Psychological stress during pregnancy predicts infant health outcomes in the first postnatal year. Matern Child Health Journal.

[58] Bogolyubova O, Pleshkova N. Prior victimization, traumatic birth experience of mothers and the quality of attachment of their young children. Eur J Psychotraumatol. 2013;4:19.

[59] Alegria M, Atkins M, Farmer E, Slaton E, Stelk W (2010). One size does not fit all: taking diversity, culture and context seriously. Adm Policy Ment Health Ment Health Serv Res.

[60] Greenbrook JTV (2016). For the development of a pluralistic and person-centered mindset among mental health practitioners. European Journal for Person Centered Healthcare.

[61] Rapley T, Flick U (2014). Sampling strategies in qualitative research. The SAGE handbook of qualitative data analysis.

[62] May T, Perry B, Flick U (2014). Reflexivity and the practice of qualitative research. The SAGE handbook of qualitative data analysis.

[63] Nunkoosing K (2005). The problems with interviews. Qual Health Res.

